# Comparison of ammonia emissions related to nitrogen use efficiency of livestock production in Europe

**DOI:** 10.1016/j.jclepro.2018.11.143

**Published:** 2019-02-20

**Authors:** C.M. Groenestein, N.J. Hutchings, H.D. Haenel, B. Amon, H. Menzi, M.H. Mikkelsen, T.H. Misselbrook, C. van Bruggen, T. Kupper, J. Webb

**Affiliations:** a Wageningen UR Livestock Research, De Elst 1, 6708 WD, Wageningen, the Netherlands; b Dept. of Agroecology, Aarhus University, Research Centre Foulum, 8830, Tjele, Denmark; c Thünen Institute of Climate-Smart Agriculture (TI-AK), Bundesallee 50, 38116, Braunschweig, Germany; d Leibniz Institute for Agricultural Engineering and Bioeconomy (ATB), Max-Eyth-Allee 100, 14469, Potsdam, Germany; e Agroscope, Inst. For Livestock Sciences, P.O. Box 64, CH-1725, Posieux, Switzerland; f Dept. of Environmental Science, Frederiksborgvej 399, 4000, Roskilde, Denmark; g Rothamsted Research - North Wyke, Okehampton, Devon, EX20 2SB, UK; h Statistics Netherlands (CBS), Postbus 24500, 2490 HA, Den Haag, the Netherlands; i Bern University of Applied Sciences, School of Agricultural, Forest and Food Sciences, Laenggasse 85, CH-3052, Zollikofen, Switzerland; j Ricardo Ltd, Gemini Building, Harwell, OX11 0QB, UK

**Keywords:** Nitrogen use efficiency, Ammonia emission intensity, Animal protein, Feed nitrogen, Manure management

## Abstract

The increasing global demand for food and the environmental effects of reactive nitrogen losses in the food production chain, increase the need for efficient use of nitrogen (N). Of N harvested in agricultural plant products, 80% is used to feed livestock. Because the largest atmospheric loss of reactive nitrogen from livestock production systems is ammonia (NH_3_), the focus of this paper is on N lost as NH_3_ during the production of animal protein. The focus of this paper is to understand the key factors explaining differences in Nitrogen Use Efficiency (NUE) of animal production among various European countries. Therefore we developed a conceptual framework to describe the NUE defined as the amount of animal-protein N per N in feed and NH_3_—N losses in the production of milk, beef, pork, chicken meat and eggs in The Netherlands, Switzerland, United Kingdom, Germany, Austria and Denmark. The framework describes how manure management and animal-related parameters (feed, metabolism) relate to NH_3_ emissions and NUE. The results showed that the animal product with the lowest NUE had the largest NH_3_ emissions and vice versa, which agrees with the reciprocal relationship between NUE and NH_3_ within the conceptual framework. Across animal products for the countries considered, about 20% of the N in feed is lost as NH_3_. The significant smallest proportion (12%) of NH_3_—N per unit of N*feed* is from chicken production. The proportions for other products are 17%, 19%, 20% and 22% for milk, pork, eggs and beef respectively. These differences were not significantly different due to the differences among countries. For all countries, NUE was lowest for beef and highest for chicken. The production of 1 kg N in beef required about 5 kg N in feed, of which 1 kg N was lost as NH_3_—N. For the production of 1 kg N in chicken meat, 2 kg N in feed was required and 0.2 kg was lost as NH_3_. The production of 1 kg N in milk required 4 kg N in feed with 0.6 kg NH_3_—N loss, the same as pork and eggs, but those needed 3 and 3.5 kg N in feed per kg N in product respectively. Except for beef, the differences among these European countries were mainly caused by differences in manure management practices and their emission factors, rather than by animal-related factors including feed and digestibility influencing the excreted amount of ammoniacal N (TAN). For beef, both aspects caused important differences. Based on the results, we encourage the expression of N losses as per N in feed or per N in product, in addition to per animal place, when comparing production efficiency and NUE. We consider that disaggregating emission factors into a diet/animal effect and a manure management effect would improve the basis for comparing national NH_3_ emission inventories.

## Highlights

• A theoretical framework describes NH_3_—N loss and NUE related to animal protein production instead of to raw product.
• Animal protein in chicken meat had the highest NUE and the lowest NH3—N losses; the reverse was true for beef.
• Differences in NUE and NH3—N losses between countries are mainly explained by manure management.
• For beef production differences in manure management and TAN production are both relevant.
• Presenting additional metrics than N-lost per animal place would enable a more informed comparison of production systems.

## Graphical abstract

**Figure:**
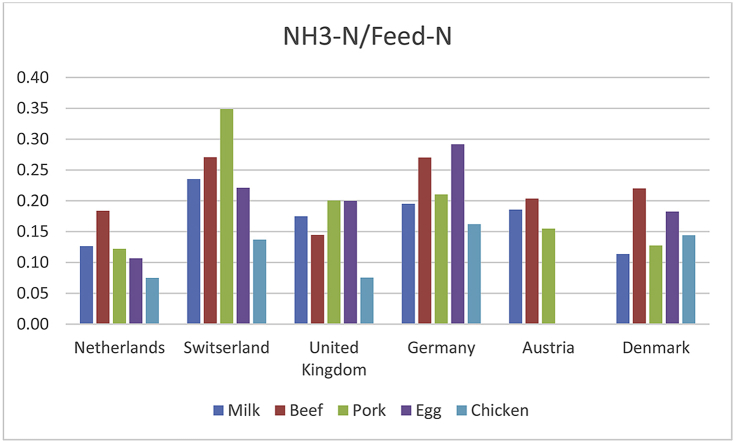

## 1 Introduction

Nitrogen (N) as a nutrient is an important contributor towards food security. With increasing world population, the demand for food increases thereby increasing the demand for reactive N. The intensification of agriculture over the last century has led to an increase in N recovery in livestock but also an increased N surplus (Bouwman et al., 2013). Inefficiencies in the production chain of food protein mean that N is lost, both as unreactive N_2_ and as reactive N compounds (N_r_), contributing the majority of the N_r_ pollution of the global environment (Bouwman et al., 2013). Ammonia (NH_3_), nitrous oxide (N_2_O), nitrogen oxides (NO_x_) and nitrate (NO_3_^−^), contribute to acidification, eutrophication and climate change, threating biodiversity, water-, air- and soil quality (Sutton et al., 2013). When deposited from the atmosphere, N_r_ cascades through a number of ecosystems (Galloway et al., 2003), with negative effects on N poor natural ecosystems in particular. Ammonia also contributes to the formation of secondary atmospheric particulate matter, with the smaller of these (PM_2.5_) implicated in a range of adverse impacts on human health.

About 80% of the N harvested in agricultural crops is used to feed livestock (Sutton et al., 2013), Managing N flows in livestock systems is therefore of critical importance when seeking to reduce N_r_ pollution. Several indicators are used to assess the efficiency of agricultural production and hence its likely contribution to environmental pollution. Nitrogen Use Efficiency (NUE), the ratio of output-N (in products) to input-N, is an indicator of the efficiency with which N input into an agricultural production system is converted to N in agricultural products. NUE can be calculated for a whole production system or for individual components (Gerber et al., 2014; Uwizeye et al., 2016). The NUE of feed (ratio of N in livestock products to the N input in feed) is one such component and is one indicator of agricultural sustainability and potential for improvement (Powell et al., 2010). Another indicator commonly used in relation to agricultural products is the N footprint (Leach et al., 2012), which is the total amount of N lost to the environment resulting from the production of a unit weight of product i.e. a measure of emission intensity. This indicator has the advantage that it includes all N inputs and losses in the food production and processing system, thereby enabling an integrated comparison for different production chains for the same product. However, it has the disadvantage that comparisons among products are difficult.

The main focus of this paper is on N_r_ lost as NH_3_ because NH_3_ is the largest atmospheric loss of N_r_ from livestock production systems (Fowler et al., 2013). In 1999, the Gothenburg Protocol of the Convention of Long Range Transboundary Air Pollution (CLRTAP) established national emission ceilings for a range of gases, including NO_x_ and NH_3_ (UNECE, 2013). This was followed in 2001 by its EU equivalent, the National Emission Ceilings Directive 2001/81/EC Directive (NECD). Ammonia emissions, of which ca. 90% originates from livestock excreta, fell by 23% between 1990 and 2015. However, the emissions of other major atmospheric pollutants fell by 50–80% in the same period. Ammonia even increased by 1.8% in the EU 28 between 2013 and 2015 (EEA, 2017), so more effort is needed to reduce these emissions.

Understanding the cause of the variation in NH_3_ emission among countries helps build confidence in the validity of national emission inventories and contributes to understanding how emission reductions may be achieved. This is an important part of periodic inventory reviews that are undertaken within the scope of the CLTRAP and NECD. The large number of parameters that influence NH_3_ emission from livestock production including animal breed, livestock diet, housing, manure management, production parameters, climatic conditions etc., makes direct comparisons of NH_3_ emissions among countries difficult. This means that at present, review teams can only calculate apparent emission factors (total NH_3_ emissions per animal category), which can identify differences among emissions reported in different national inventories but not their origin. The main livestock products consumed in Europe are dairy (cheese, milk), pork, poultry meat, beef and eggs (EEA, 2017), so the categories for the production are dairy and beef cattle, pigs, laying hens and broilers. Common to these is the conversion of feed protein into animal protein. Ammonia-N expressed per unit product or per N in product enables a direct comparison among different animal products, provides a better insight regarding the differences among countries and thus insight regarding the mechanisms of efficiency parameters of protein turnover. Therefore this paper compares NH_3_—N emissions among various European countries in relation to the production of animal protein from feed protein. The methodology is developed and implemented for the production of milk, beef, pork, chicken meat and eggs in The Netherlands, Switzerland, United Kingdom, Germany, Austria and Denmark. Using a conceptual framework of the N-flow in livestock systems, this paper aims to establish which parameters we need to focus on, distinguishing animal-related factors (quality and quantity of the feed, protein turnover and consequent NUE) on the one hand and manure management, explaining the fraction of total ammoniacal N (TAN) that emits as NH_3_—N, on the other.

## 2 Material and methods

### 2.1 Conceptual framework

**Fig. 1:**
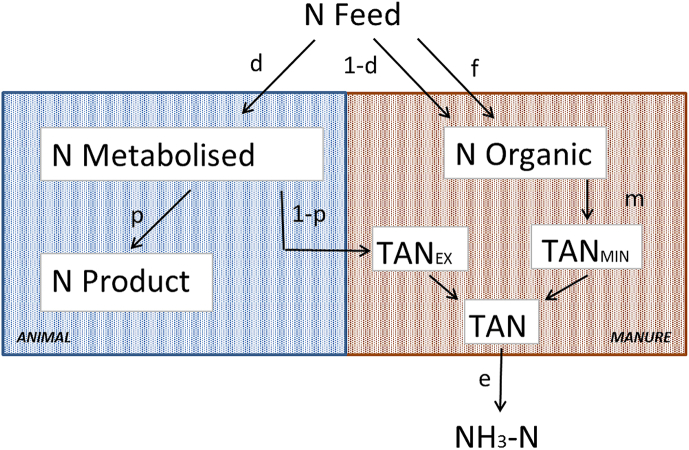
Conceptual framework of how nitrogen in feed (N Feed) is transformed into emission of nitrogen as ammonia (NH_3_—N) and nitrogen in product (N Product); *d* = apparent digestibility of feed, p = proportion of N metabolised deposited in product, f = feed waste, m = fraction of organic N in manure mineralised to TANmin (Total Ammoniacal Nitrogen mineralised), TANex = TAN excreted, e = fraction of total TAN emitted as NH_3_—N.

A conceptual framework was developed of NH_3_ emissions from livestock and manure management (Fig. 1). This framework represents a simplification, since manure management is considered as a single entity, whereas in practice, it comprises several elements (e.g. livestock housing, manure storage). The source of the volatile NH_3_ in manure is TAN. The production of TAN from N in feed is shown in Fig. 1. A proportion *d* of the feed N (*Nfeed*; kg day^−1^) is digested and absorbed by the livestock, yielding an amount of metabolisable protein. A proportion *p* of the metabolisable protein is then converted to N in milk, meat or eggs. For milk, the whole of the product is of value for human nutrition and this is essentially also true for eggs, since the shell forms a small part of the product. For beef and pork, the inedible part of the carcass (mainly bone) is significant. However, this study was a comparison of the environmental impacts of raising different types of livestock, rather than of individual livestock end products. Moreover, since bones etc. are needed to produce the edible portion of the carcass, we considered it appropriate for this study that the N in the whole carcass should represent the product N. The N retained in animal protein (*Nproduct*; kg day^−1^) is therefore:

(1) Nproduct=d∗p∗Nfeed

And the NUE of feed (NUE*feed*) is:

(2) NUEfeed=d∗p

The remainder (1-*p*) of the N in metabolisable protein is excreted to the urine (milk, beef and pork production) or in droppings (poultry), and can be considered to be TAN (TANex). A part of the feed protein will normally be indigestible and a proportion of the digestible feed protein is used to form microbial protein in the gut. These are excreted as organic N in the faeces. Organic N also enters manure through spilled feed, defined as *f*. A proportion (*m*) of the N in manure can be mineralized (TANmin), and thus also contributes to the amount of TAN. The TAN produced (*TAN*; kg day^−1^) is therefore:

(3) TAN=Nfeed(d(1−p)+m(1−d+f))

A proportion of the TAN (*e*) volatilizes as NH_3_—N from manure in livestock buildings and manure stores, following manure application to land and from excreta deposited during grazing. The masses of N_2_O—N and N_2_—N emitted are much less than that of NH_3_—N hence they are omitted here. The value of *e* depends on numerous factors, of which livestock type, temperature, air velocity, pH and concentration of TAN are the most important. The NH_3_—N emission is therefore:

(4) NH3N=e∗Nfeed(d(1−p)+m(1−d+f))

The NH_3_ emission intensity (AEI), which is the NH_3_—N emission per unit of N in the product (kg kg^−1^) can be calculated from (Eq. (5)):

(5) $$AEI=\frac{NH3N}{Nproduct}\;\;=\;\;\;\frac{e(d\left(1-p\right)+m\;\left(1-d+f\right))}{d*p}$$

From Equation (2), Equation (4) can be written as Equation (5):

(6) $$AEI=\;\frac{e\;(d\left(1-p-m\right)+m\left(1+f\right))}{NUEfeed}$$

This equation shows that the *AEI* will be related to the inverse of the NUE*feed* (further referred to as NUE) and that the nature of that relationship will depend on a combination of feed/animal characteristics (*d*, *p*; i.e. TAN excreted) and manure management system characteristics (*f, m, e*).

**Fig. 2:**
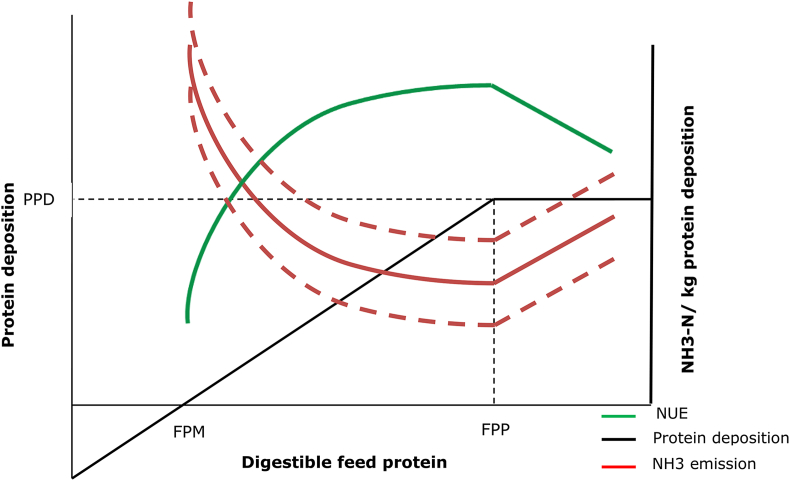
Protein deposition, nitrogen use efficiency (NUE) and ammonia (NH_3_—N) emission related to digestible protein in feed. FPM and FPP are digestible feed protein at maintenance level and potential production level respectively. The solid red line is NH_3_—N emission per kg protein deposition (EI), the solid green line is NUE (no dimension), and dotted red lines are lower or higher NH_3_—N emission due to factors related to manure management.

Fig. 2 represents protein deposition given sufficient energy supply. When the metabolisable protein (MP) supplied in feed is insufficient to satisfy the animal's maintenance protein requirement, protein will be remobilised from body tissues, the animal will lose weight and no protein deposition in egg, milk or meat will occur. With increasing MP intake, protein deposition increases until the maximum (potential) protein deposition (PPD) is obtained (Whittemore and Fawcett, 1976). The efficiency with which the MP in excess of the maintenance requirement is used for protein deposition varies, depending on the quality of the MP (in particular, the balance of essential amino acids) and on the type of protein (egg, milk, meat) being deposited. The animal's genetic capacity limits PPD. Ideally this occurs with a feed intake supplying the minimum protein needed for potential protein deposition (FPP). Above FPP, surplus feed protein N is all lost as TAN in urine, illustrated by the linear relationship of NH_3_ and NUE with increasing feed protein content in Fig. 2. The figure illustrates firstly that by decreasing FPP by maximizing *p* while avoiding protein in feed beyond FPP minimizes NH_3_—N emission per kg product-N. Secondly, NH_3_ emission will decline if *e* is reduced. This will lower the NH_3_ curve parallel to the y-axis. Finally, *m* should be minimized during storage. In practice, digestibility of N*feed* in intensive livestock systems only varies between about 0.7 and 0.9. However, for cattle on extensive grassland, lower digestibility feeds are encountered, especially outside the growing season (Armstrong et al., 1986).

If *e* was equal, differences among countries would arise through: genetic potential of maintenance requirement (MR); genetic potential of PPD; FPP; actual feed protein. The amount of protein in the feed, FPM and FPP depend on inherent genetic properties, but also on feeding management, including protein quality and essential amino acids (Fig. 2).

The main manure-related factors influencing *m* and *e* are: surface area; temperature; air velocity; pH (Webb et al., 2012). Ammonia emission is also related to manure management: how manure is stored; where it is stored; for how long. Some countries have introduced emission reduction techniques. Variation of these aspects within the theoretical framework will move the red line up parallel to the Y-axis (high *e*), or down (low *e*) (dotted red lines).

### 2.2 Data analysis

The national emission inventories of The Netherlands, Switzerland, United Kingdom, Germany, Austria and Denmark were used to estimate NH_3_ emission from livestock production. These models and data regularly undergo a detailed external review for quality control and quality assurance and were tested for congruency by Reidy et al. (2008, 2009). The principal mass flow approach compared well among the national models when all use the same input values for TAN excretion (following *d* and *p*) and NH_3_ emission factor (*e*).

Data were collected from the six countries for dairy, beef, fattening pigs, laying hens and broilers. For Austria poultry production is of minor importance and was excluded from the analyses. The data used for each livestock category were: N intake and excretion; percentage of TAN in excreted N; the NH_3_ emission factors (% of TAN) at housing, manure storage, during grazing and from manure after field application. To express in relation to N in product, data were collected on growth, milk production and egg production as well as the N-content of livestock animals, eggs and milk. The N contained in meat was defined as the N-content of the ground carcass. For dairy, only milk production was included, not the meat of the cull cow after milk production or of the calves produced during the cow's lifetime. For eggs the shell was included but the meat at the end of the laying cycle was not. Parental animals or young replacement animals were not considered. The only N-input therefore was the N in feed. The N-content of the products was calculated as the mean of the national data, apart from Austria, for which there were no national data and Swiss values were used.

The data were used to calculate NUE as N*product*/N*feed*. The NH_3_—N lost was expressed per N deposited in product. The implied emission factor (IEF) was calculated as the sum of all NH_3_—N-emissions over the manure management chain, expressed as % of TAN.

Annual NH_3_ emission can be considered as the product of the processes determining the amount of TAN that is susceptible to volatilisation and the IEF, with the former described in Equation (2). The component *d*(1-*p*) describes the processes that take place within the animal while the component *m*(1-*d*) describes those taking place in the manure management chain (Fig. 1). The factor *m* can take a positive or negative sign; in some manures, mineralisation of organic N leads to the formation of TAN whereas in others, there is an immobilisation of TAN into organic N (Jensen and Sommer, 2013). Not visualised in Fig. 1, but influencing *m*, is the amount of bedding material, affecting *m* through availability for microbial energy needs, but also adding a small amount of N to the manure. Not all countries take *m* into consideration in their NH_3_-inventory model (Reidy et al., 2009). Some countries (e.g. DK, NL) have made more effort to reduce NH_3_ emission, resulting in a variety of abatement techniques for housing, storage and field application, further contributing to variation in manure management. NH_3_—N losses per kg N in feed for all animal products were assessed and compared using a double sided two-sample Student's t-test with *P* < 0.05.

The origin of the variation in emission intensity within a product can be explored by separating out the contribution TAN excretion makes to NH_3_ emission (NH3_TANex_) from that made by the manure management system (MMS) (NH3_MMS_). For any dataset containing estimates of NH_3_—N emission and TAN excretion, we can calculate the mean NH_3_—N emission (NH3_ave_) and TAN excretion (TANex_ave_). Using the subscript *i* to identify an individual element in the dataset, then:

(7) NH3_TANexi_ = (TANex_i_*(NH3_ave_/TANex_ave_)—NH3_ave_)/NH3_ave_

(8) NH3_MMSi_ = (TANex_ave_*(NH3_i_/TANex_i_)—NH3_ave_)/NH3_ave_

The sum of NH3_TANexi_ and NH3_MMSi_ expresses the extent to which the NH_3_—N emission from element *i* deviates from the mean of the dataset. If plotted, a quadrant is drawn with clockwise a: low NH3_TANex_, high NH3_MMS_; b: high NH3_TANex_, high NH3_MMS_; c: high NH3_TANex_, low NH3_MMS_; d: low TANex, low NH3_MMS_

## 3 Results

Table 1 presents for each country per animal place and year the total amount of N fed, the excretion of TAN by the animal, the IEF as percentage of excreted TAN and the production of N in the animal product (egg, milk or meat). NUE of the feed did not differ as much as TAN and IEF due to less variability of N*feed* and N*product*, with a coefficient of variation (cv) of 5–14%. The IEF had the largest cv varying between 27 and 39% with the smallest variation for milk and pork, and the largest for chicken.

**Table 1:** Feed-N, TAN excretion, implied emission factor of NH3—N (IEF), N production in kg/y per animal place and Nitrogen Use Efficiency of the feed (NUE) defined as N production/Feed-N, CV is coefficient of variation.

		Netherlands	Switserland	United Kingdom	Germany	Austria	Denmark^b^	Mean	CV, %
Milk	Feed-N	176	150	153	155	124	189	158	14
	TAN excretion	84	62	72	64	58	64	67	14
	IEF a	27	58	37	47	39	34	40	27
	N production	44	39	40	39	33	50	41	10
	NUE	0.25	0.26	0.26	0.25	0.27	0.26	0.26	11
Beef	Feed-N	47	46	67	52	56	44	52	17
	TAN excretion	23	20	34	26	24	22	25	19
	IEF a	38	64	17	54	50	41	44	36
	N production	11	10	15	10	10	11	12	16
	NUE	0.23	0.23	0.22	0.20	0.17	0.24	0.22	5
Pork	Feed-N	19	17	20	19	20	19	19	5
	TAN excretion	8.4	8.5	8.4	9.1	6.7	7.0	8.0	12
	IEF a	27	63	47	45	45	35	44	28
	N production	7.4	6.4	5.9	6.2	6.4	7.4	6.6	11
	NUE	0.39	0.37	0.30	0.32	0.33	0.39	0.35	12
Egg	Feed-N	1.09	1.31	1.14	1.21	–	1.15	1.18	7
	TAN excretion	0.57	0.48	0.47	0.58	–	0.53	0.52	10
	IEF a	21	59	49	61	–	40	46	36
	N production	0.33	0.33	0.34	0.35	–	0.37	0.34	6
	NUE	0.30	0.25	0.29	0.29	–	0.32	0.29	9
Chicken	Feed-N	0.9	0.9	1.0	1.28	–	1.0	1.0	16
	TAN excretion	0.28	0.27	0.28	0.33	–	0.30	0.32	20
	IEF a	23	45	28	63	–	50	46	39
	N production	0.45	0.44	0.54	0.61	–	0.58	0.54	14
	NUE	0.52	0.51	0.52	0.48	–	0.55	0.55	14

a IEF is total emission of NH3—N from housing to application as % of excreted TAN.

b Danish data for solid manure are not based on TAN, but on N, TAN assumed is the average of NL, CH, UK, GE and A with 60%, 67%, 62% of Nexcretion for beef, laying hens and broiler respectively.

The N*product* of milk and egg are easy assessable and frequently monitored, especially for milk because the market price depends on the protein content, so nationally-derived statistics were used for these data (Table 2). The N*product* of meat is costly to assess and not routinely measured. When measured, it is the N-content of the carcass, which for this study is considered the same as the N-content of the meat. The mean of values given by the NL, CH, UK, DE and DK were used for these data rather than national-specific values (Table 2). The variation coefficients were relatively low except for the protein content of the chicken due to the low N-content of UK chicken.

**Table 2:** Protein content of animal products (g/kg product) of The Netherlands, Switserland, United Kingdom, Germany and Denmark, the mean and the coefficient of variation (CV), %.

Product	Netherlands	Switserland	United Kingdom	Germany	Denmark	Mean	CV,%
Milk	35.2	34.4	33.0	34.1	34.7	34.28	2.4
Beef	169	175	154	170	164	166.4	4.8
Pork	163	156	164	160	169	162.4	3.0
Egg	116	113	125	119	113	117.2	4.3
Chicken	174	182	147	190	180	174.6	9.4

**Fig. 3:**
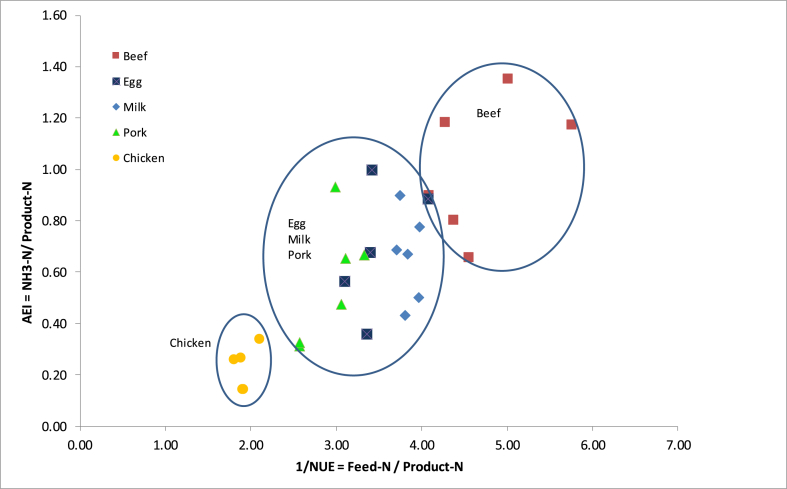
Relationship between AEI (NH_3_—N/N in product) and NUE for 5 livestock products of 6 European countries (The Netherlands, Switzerland, United Kingdom, Germany, Austria and Denmark). (For interpretation of the references to colour in this figure legend, the reader is referred to the Web version of this article.)

Fig. 3 shows the reciprocal effect between NUE and NH_3_—N emission per N*product*. In terms of NUE the order from low to high was: beef 0.22; milk 0.26; eggs 0.29; pork 0.35 and chicken 0.55. For NH_3_—N this was chicken 0.27, pork 0.53, milk 0.65, egg 0.70 and beef 0.92. The product with the lowest NUE had the largest NH_3_ emissions and vice versa, which agrees with the reciprocal relationship within the theoretical framework shown in Fig. 1, Fig. 2. For all countries, NUE was lowest for beef and highest for chicken, with the production of 1 kg N in beef requiring about 5 kg N in feed, of which about 1 kg N was lost as NH_3_—N. In contrast, the production of 1 kg N in chicken meat required about 2 kg N*feed* and 0.2 kg was lost as NH_3_. The production of N in milk required ca 4 kg N*feed* with ca 0.6 kg NH_3_—N loss, the same as with eggs and pork, but those needed only 3 kg N*feed* per kg N in product. From Equation (6), we note that the slope of this relationship will be *e* (*d*(1-*p*)+*m*((1-*d * + * f*)) and if extrapolated, it will intersect with the x-axis where NUE = 1 (theoretical point where all the N*feed* is deposited in the product and no NH_3_ emission occurs). Furthermore, we note that since N*product* appears in the definition of both AEI and NUE, the relationship AEI versus 1/NUE is identical to NH_3_—N versus N*feed*.

Table 3 gives the NH_3_—N for every kg N that is fed to the animal. It shows that the NL chicken meat has the lowest environmental impact because only 8% of the N-input is lost as NH_3_—N. Swiss pork is highest because 35% of the N*feed* is lost as NH_3_—N. Variation among countries is large, the data do not provide enough information to significantly discriminate among animal products, except for the difference between beef and chicken.

**Table 3:** NH_3_—N losses per kg N in feed for all countries and animal products, the mean per product and the variation coefficient (%). For the last column, different letters mean significant difference between rows based on two-sample t-tests.

	Netherlands	Switserland	United Kingdom	Germany	Austria	Denmark	Mean	CV,%	P < 0.05
Milk	0.13	0.24	0.17	0.20	0.19	0.11	0.17	27	ab
Beef	0.18	0.28	0.14	0.27	0.20	0.22	0.22	24	a
Pork	0.12	0.35	0.20	0.21	0.15	0.13	0.19	43	ab
Egg	0.11	0.22	0.20	0.29	–	0.18	0.20	33	ab
Chicken	0.08	0.14	0.08	0.16	–	0.14	0.12	34	b

**Fig. 4:**
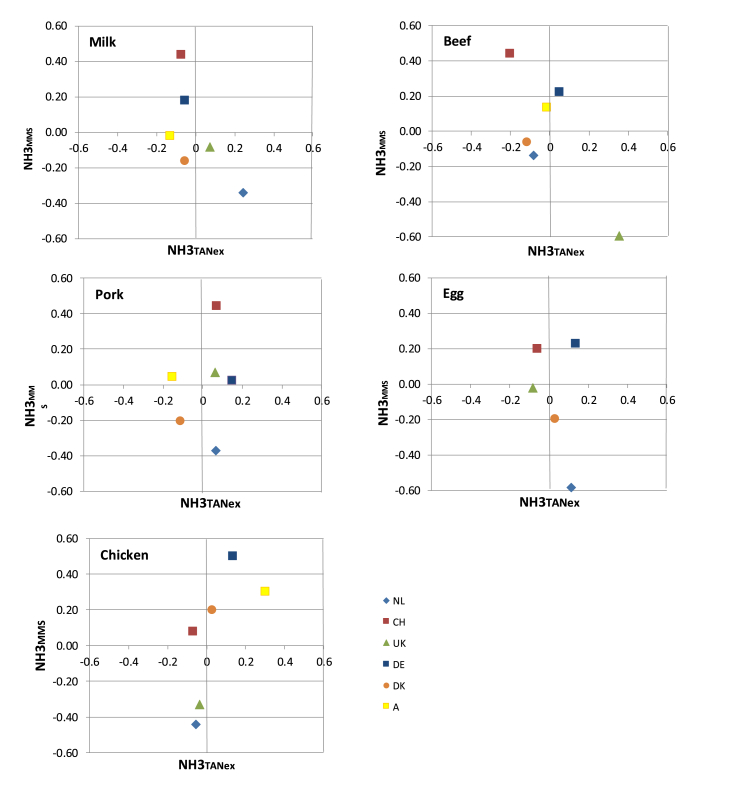
Relative contribution of manure management (EF) to NH_3_ (NH3_MMS_) versus that of TAN excretion (NH3_T__ANex_) for milk, beef, pork, eggs, and chicken in the six different countries NL (light blue), CH (red), UK (green), DE (dark blue), A (yellow), DK (orange); a: low TAN, high EF; b: high TAN, high EF; c: high TAN, low EF; d: low TAN, low EF. (For interpretation of the references to colour in this figure legend, the reader is referred to the Web version of this article.)

Fig. 4 plots NH3_MMSi_ against NH3_TANex_ from which the relative contribution to NH_3_—N emission can be seen. For all animal products, the differences among countries in MMS are larger than in TANex. For chicken, the differences in TANex are smallest and for beef largest. Livestock products from the NL are always in quadrant c or d indicating the lower emission factors by MMS. The results for DK are also in c and d except for chicken, which are in quadrant b (relatively high for both TANex and MMS). The converse is the case for CH and DE, they are always in a or b, with highest MMS emissions. Noticeable is the exceptional position of UK beef in quadrant c with very high TANex and at the same time low MMS emissions. The opposite is the case for CH in quadrant a. Secondly it is noticeable that UK has quite low MMS emissions for chicken. The small MMS emissions for UK beef is due to the animals grazing all day for ca 6 months of the year and depositing ca 55% of their annual N excretion to grassland. Ammonia emissions from excreta deposited on grazed pastures are only about 20% of those from excreta deposited in and around buildings and handled as manure. This is because the N in urine is predominantly in an organic form (mainly urea) and most of this infiltrates rapidly into the soil, before hydrolysis to TAN can occur. The small MMS emissions for broilers is because emissions from UK broiler buildings, estimated as 10% of TAN excreted, are less than those estimated for the other countries.

## 4 Discussion

The reciprocal of NUE reflected the differences in AEI (Fig. 3). Although differences among countries were large, beef, with the lowest NUE had the highest NH_3_—N loss per kg N*product* and chicken meat with the highest NUE, the lowest. Since N*product* appears in the definition of both AEI and NUE and the relationship AEI versus 1/NUE is identical to NH_3_—N versus N*feed* we encourage the expression of N losses per N in feed or N in product, in addition to the loss per animal place, when comparing production efficiency and NUE.

The large losses from beef production and the small losses from chicken production correspond with the results of Leip et al. (2014) who calculated the N footprint with the Nitrogen Investment Factor (NIF) of different food products, defined as kg N-input per kg N product at the farm level. For beef, they calculated 15–20, compared with 3–5 kg N/kg N for chicken, pork, milk and eggs. This is equivalent to 1/NUE in this study which was 5 for beef, and 2–4 kg/kg for chicken, pork, eggs and milk. The differences in between our findings and those of Leip et al. (2014) occur primarily because we limited this study to the N flows in the animals and MMS, so did not include soil processes. However, it is possible the gaseous products associated with the denitrification process (N_2_O, NO and N_2_) were relatively more important for beef systems. This would be consistent with the greater N_2_O emission factor (2.0%) used for IPCC for N deposited during grazing than for N applied to soil in manure (IPCC, 2006). We also limited our study to the N input in feed for adult animals and did not include the animals reared for replacement. An additional factor is that the protein contents in Table 2 are measured as that of the ground carcasses. The greater proportion of non-edible product in beef production compared with poultry meat means that our approach increases the proportion of feed N captured in the beef. This study did not take inputs from litter into account, which are an additional N source for dairy and beef especially. For dairy cows, for example, 1.5 kg of straw per cow per day would increase the organic N input compared with N*feed* by ca 5%. A fraction would be converted into TAN, depending on the net mineralization, being the sum of mineralization and immobilization of organic N. As Webb et al. (2012) describe, this depends on various factors including temperature and the availability of oxygen, which depend on manure management. Overall, it was considered that omitting the litter N from the calculations had little impact on subsequent results and interpretation. Leip et al. (2014) also considered crop growth in their meta-analysis. Yet the similarity of the results for chicken, pork, milk and eggs is remarkable, indicating that NH_3_ is the major N component of the N footprint. Finally, the fact that we did not take into account the meat production of the milked cow, can affect balances among the environmental impact of products. In terms of the ranking of livestock products according to the environmental impact per unit of product, our findings are consistent with LCA studies in which eutrophication impacts are expressed per kg of the most economically important fraction(s) of the carcass; i.e. beef has the greatest impact per kg and poultry meat the smallest (e.g. De Vries and De Boer, 2010, Williams et al., 2006).

The breakdown of feed-protein into NH_3_—N as shown in Fig. 1 gives the NH_3_-emission per kg N*product* as presented by the red solid line in Fig. 2. From Equation (6) we know the reciprocal relation between NH_3_ and NUE, this is visualized in Fig. 2 with the solid green line. When feed protein > FPP, NUE decreases because *p* decreases and with increasing amount of digestible protein (*d*), TAN increases linearly. With equal urine volume, this means an increased concentration of TAN, resulting in a linear increase of NH_3_ emission. If the animals are fed to supply their specific protein demand, they will be fed at FPP. With phase feeding and the addition of supplementary amino acids, farmers try to feed closer to FPP. However, this is expensive and to be safe and ensure the genetic potential of production is realised, farmers will often feed above this level, using lower cost formulations that ensure essential amino acid requirements are met but others may be exceeded.

Table 3 gives the NH_3_—N for every kg N that is fed to the animal. With livestock being the main source of NH_3_—N and NH_3_—N being the main atmospheric Nr of livestock production, this factor gives a good impression of the acidifying impact of a system within the identified boundaries, in this case from the N in the feed to the N on the field. The results of the LCA review of De Vries and De Boer (2010) showed less impact on acidification (i.e. NH_3_ emissions) for milk and egg production than for meat products. However, they expressed the impact per kg product which in the case of milk and eggs contain more water and are therefore hard to compare with meat. In the case of this study the impact of milk would then have been ca. 5 times less, and of eggs, ca 1.5 times (Table 2). Expressing N-losses per N*product* is a better measure for N efficiency or N recovery when comparing across products. The variation in this study was much smaller than the 80% observed by De Vries and De Boer (2010), nevertheless, the data did not provide enough information to significantly discriminate among products, except for chicken and beef (Table 3). To explain differences among countries we have to observe the different processes as described theoretically in Fig. 1, Fig. 2 and the calculated results in Fig. 3. These results indicate that to explain differences among countries for NH_3_ emissions due to production of pork, chicken, milk and eggs, priority should be focussed on the differences in MMS among countries, rather than on the TANex. Fig. 4 visualizes this more closely. For beef, TANex might be an interesting factor to observe more closely, although given the high IEF (Table 1) the emission factors should not be neglected. A low NUE for grazing livestock is not necessarily an environmental problem: i. because these livestock convert plant material that is inedible by humans into meat and milk, and ii. because the NH_3_—N emission per N*feed* is still acceptable, since the NH_3_ emissions from excreta N deposited during grazing are much lower than from excreta N flowing in manure management systems. Understanding the differences brings us closer to reducing the environmental impact of livestock.

The results in Fig. 4 imply that, with the exception of beef production, the differences in NH_3_ emissions among countries are mainly a result of manure management factors, rather than due to differences in TAN excretion. With beef, large differences are caused by TANex as well. Theoretically variation in TANex depends on genetic potential of maintenance requirement (MR), genetic potential of protein deposition (PPD), the amount of feed protein at maximum protein deposition (FPP) and the actual feed protein (Fig. 2). For the countries considered in this study, the differences in animal genetics and in diet formulation are probably less than differences in manure management practices (particularly considering the uptake of ammonia emission mitigation techniques). A more global study including a much greater diversity in systems may reveal larger differences in genetics and diets.

A low NH3_MMS_ (quadrant d and c) in Fig. 4 reflects efforts made to achieve emission reductions. In the NL and DK reduced emission manure application is mandatory and reduced emission housing for pigs is standard procedure. Also, reduced emission poultry housing is standard in NL, and only a small amount of the poultry manure is applied to land, because it is burned for electricity or exported. Denmark has a high NH3_MMS_ for poultry (quadrant b) because it is just a small market and the effort to invest in reduced emission buildings would have little impact at the national scale. It should be noted that differences among countries are not implying good or bad in terms of agricultural sustainable performance. For instance, animal welfare considerations in CH demand that livestock have access to a floor area larger than in other countries and this leads to larger emissions and values end up in quadrant a or b. In contrast, the NH_3_—N emission for beef in the UK in quadrant c, is below the mean of the dataset. Here, beef production is mainly pasture-based, which results in the TAN excretion being above the mean of the dataset. However, this is outweighed by the much lower NH_3_ emissions from the excreta deposited on pasture than from housed animals. Secondly, as Norton et al. (2015) noted, interpretation of NUE gives an idea of potential for improvement. Another context might give another view on efficiency as illustrated by OECD (2008) using gross N-balances as agro-environmental indicators referring input of N (volatile N compounds, fertilizer-N, manure-N, biological N-fixation and atmospheric N-deposition) and output of N (arable crop-N, fodder crop-N and pasture-N) resulting in the highest NUE for CH (0.6) and the lowest for DK and NL (0.4–0.45) in 2008, giving another view on improvement. In this context, reducing NH_3_ emission would not improve efficiency, because the lower N input of NH_3_—N would be completely compensated by the higher manure-N input. In our study NH_3_—N reduction is a key factor to improve NUE and the environmental impact of livestock production.

## 5 Conclusions

Across animal products for the countries included in the study, about 20% of the N in feed is lost as NH_3_. For chicken meat NH_3_—N/N*feed* is significantly lowest at 12%. For the other products NH_3_—N/N*feed* are 17%, 19%, 20% and 22% for milk, pork, eggs and beef respectively, these were however not significant due to differences among countries. To produce 1 kg of N in chicken, pork, eggs, milk and beef, 2, 3, 3.5, 4 and 5 kg of N in feed are needed respectively.

NH_3_—N/kg N*product* (Ammonia Emission Intensity) and NH_3_—N/N*feed* are considered better metrics to compare the environmental impact of livestock than NH_3_—N emission per animal place or per kg product and are good indicators for assessing the efficiency of potential mitigation measures. At the same time, high emission intensities may reflect trade-offs with animal welfare and with the conversion of human-inedible protein sources (e.g. forages) to edible animal products by ruminants.

On the basis of the conceptual framework presented for NH_3_ emissions from livestock production systems, it appeared that the larger part of the differences among countries were caused by differences in manure management practices and their emission factors, rather than by TANex and feed digestibility parameters, except for beef where both aspects are of importance.

The expression of N losses from animal production is assisted by using NUE as an indicator. Furthermore, the disaggregation of the emissions into a TAN effect and an effect of the manure management system is a useful way to help understand differences in emissions among national NH_3_ emission inventories and form the basis of a discussion during the periodic NH_3_ inventory review.
